# Infrared Thermography Approach for Pipelines and Cylindrical Based Geometries

**DOI:** 10.3390/polym12071616

**Published:** 2020-07-21

**Authors:** Saed Amer, Houda Al Zarkani, Stefano Sfarra, Mohammed Omar

**Affiliations:** 1 Department Industrial and Systems Engineering, Khalifa University, Abu Dhabi 127788, UAE; saed.amer@ku.ac.ae (S.A.); houda.alzarkani@ku.ac.ae (H.A.Z.); mohammed.omar@ku.ac.ae (M.O.); 2 Department of Industrial and Information Engineering and Economics, University of L’Aquila, AQ 67100 L’Aquila, Italy

**Keywords:** complex geometries, nondestructive testing, infrared thermography, line scan thermography (LST), signal to noise ratio (SNR), analysis of means (ANOM), analysis of variance (ANOVA), aspect ratio (AR)

## Abstract

Infrared thermography (IRT) is a competitive method for nondestructive testing; yet it is susceptible to errors when testing objects with complex geometries. This work investigates the effects of regulating different thermographic testing parameters to optimize the IRT outcomes when testing complex shaped geometries, particularly cylindrical coupons. These parameters include the scanning routine, feed-rate, and heat intensity. Fine-tuning these parameters will be performed with respect to three different variables consisting of workpiece density, defect size, and defect depth. The experimental work is designed around 3D-printed cylindrical coupons, then the obtained thermal images are stitched via image processing tool to expose defects from different scans. The analysis employs a Signal-to-Noise Ratio (SNR) metric in an orthogonal tabulation following a Taguchi Design of Experiment. Moreover, test sensitivity and the best combination of factor levels are determined using Analysis of Means (ANOM) and Analysis of Variance (ANOVA). The outcomes show that the heating intensity factor is the most dominant in exposing flaws with close to 40% mean shift and up to 47% variance fluctuation. The paper introduces the tools employed in the study, and then explains the methodology followed to test one sample quadrant. The results for running the testing on all the scenarios are presented, interpreted, and their implications are recommended.

## 1. Introduction

Quality inspection is a vital but challenging process. Manual inspection tends to be inclusive but exhaustive and prone to human errors. Automatic inspection, on the other hand, may not be comprehensive, as it may be limited by the adopted technique. Nonetheless, internal, and inconspicuous defects may be more challenging, as conventional inspection methods have limited subsurface penetration. Such, conventional destructive methods may also require specialized experts, thus, limiting their industrial adoption [1]. Conversely, nondestructive testing (NDT) methods improve the inspection quality by detecting embedded defects without altering the material structure or geometrical shape [2,3]. Infrared Thermography (IRT) offers great potential due to its contactless nature, and fast inspection rates [4]. Nonetheless, IRT inspection routines are still sensitive to the size, the shape, the material thermal properties, and lastly the depth of the defects [5]. More precisely, complex-shaped objects may pose more challenges for thermal imaging due to optical and emissivity issues. Some of these challenges include irregular heat distribution, emissivity variations, and other radiation-based complications (shape factor) due to line-of-sight nature. Zhu et al. affirmed that artifacts with several embedded components are more challenging to inspect because, of the different emissivity values at the same surface (or Field of View); hence, the thermal image may not be accurate [6]. Furthermore, the specimen’s emitted radiation to the detector can be affected by environmental, thermal noise, contributions such as background reflection, and contributing media [7].

Line Scan Thermography (LST) is a scanning routine that operates by passing a heating source and an infrared detector to scan a specimen in real time [8]. Robotized versions of the LST techniques offer new capabilities in terms of accuracy/consistency of the scanning head parameters, which might be essential for optimizing the thermography detection. In other words, a robotized LST provides enhanced detection probability by maintaining the heating uniformity, while reducing the cost of inspecting complex objects and surfaces [9]. Khodayar et al. proposed a novel systematic algorithm to optimize such parameters including scanning speed, source power, heating intensity, and other parameters in [10]. A 3D finite element simulation is used to investigate the optimal parameters of a specimen, made of a porous and anisotropic structure. The results of the referred study showed improved detection and accuracy of the depth at a higher Signal-to-Noise Ratio SNR [10].

Vavilov et al. discussed different test procedures of Thermography NDT to detect defects in cylindrical parts. The study employed one-sided and two-sided tests with three scanning approaches including spot heating, uniform heating, and line heating. The spot and line heating approaches are carried out while the specimen is rotating and being subjected to a heating source. According to the results obtained from the experiments, uniform and line heating modes managed to detect artificial defects at a depth of 1.64 mm given a 4 mm depth detection limit [11]. Peeters et al. performed a study on a bicycle frame of Carbon Fiber Reinforced Plastics (CFRP), aiming to check for defects using active thermography. The experiment is composed of a six-axis robot equipped with an infrared camera and an excitation source [12]. Further studies pushed on to employ a statistical method called Probability of Detection (POD) to estimate the proportion of defects with line scan thermography detection. The proposed work shows that as the POD decreases and the complexity of the samples increases the accuracy and scanning speed are significantly enhanced [13]. In another study, the optimal locations of the excitation and measurement points were determined by incorporating both the robotic arm and an advanced path planning tool. Hence, numerical simulation is used to interrogate the optimal experimental parameters, to efficiently detect defects using active thermography. COMSOL^®^ Multiphysics 5.0 software (Stockholm, Sweden) was used to generate the numerical model and then correlate the results with the NDT data [14].

Eitzinger et al. presented a study on the parameters associated with calculating the path on a 3D object such as the optimal distance between the camera and the specimen while ensuring the maximum field of view; in this study, the path planning develops a set of viewpoints covered by the robot [15]. Obtaining a maximum coverage of viewpoints could be similar to the “greedy-generate and test” algorithm used by robotizing the in-line scanning experiment in [9]. According to Ng et al., there are several parameters to be identified before running an NDT experiment; some are controllable, and some are driven by noise factors [16]. The controllable factors are the ones that can be manipulated during the experiment and have direct effects on the process outcomes. The signal factor has an average effect on the response value, while the noise factor has a complete effect. Thus, altering these factors using high, low, and medium levels will allow the capturing and analyzing of different responses [16].

Different numbers of factors and levels are considered for different experiments. After designing the experiment and identifying the factors and levels for each factor, a Taguchi-method with orthogonal arrays exploited for the experiment design and testing campaign. An orthogonal array is a standardized approach that enables the determination of the number of experiments to be conducted and in which combination [17]. After analyzing the results obtained from the experiment, the SNR ratio can be calculated. According to Chen et al., there are three different perspectives on the signal-to-noise ratio: the smaller the better, the larger the better, or the more proximal to a nominal value the better [17]. In addition, two more statistical analyses are considered in order to test the sensitivity of detection: one probes the population averages, i.e., analysis of means (ANOM), while the second one is called analysis of variance, which evaluates the consistency of factors (ANOVA) [18]. While typical IRT testing is done per usage protocol or standards with simple flat geometry, this work employs a design of experiment for infrared inspection routines coupled with unique cylindrical geometries. Three major control factors are manipulated while observing four measurable outcomes that quantify the detectability of hidden defects. Furthermore, the testing is carried out on 40 different defects with varying sizes, shapes, depths, and material densities to ensure outcome validity. Employing finite element analyses simulation and validate it with 3D-printed test coupons complements to the novelty of the work. The presented study demonstrates a testing campaign and a procedure that can be duplicated under different sample conditions.

Section 2 provides the steps followed to perform the experimentation along with the needed equipment, materials, and setups. Section 3 presents the results obtained by running the experimentations seeking to test the different factors that impact the detectability of hidden defects with IRT. Section 4 discusses the interpretation of the results and their implication on the testing methodology. Ultimately, Section 5 presents the conclusions made from modifying the designated factors in the performance of the Infrared Thermography testing.

## 2. Materials and Methods

**Figure 1:**
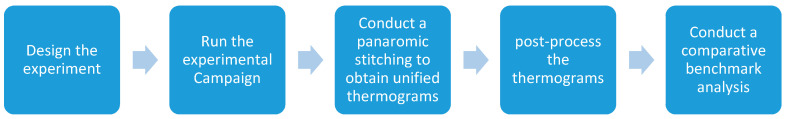
Methodology flow chart.

The methodology of the proposed research follows a step-by-step systematic approach that ensures feasible and effective investigation as shown in Figure 1. The testing will feature an automated setup in addition to a manual one. The design of the experiment includes a set of controllable factors such as the speed of the automated arm and the heating intensity. On the other hand, the fixed-scanning experiment controllable factors include the speed of the servo motor and the heating intensity. The number of experiments conducted, and the different combinations are determined by the standardized table of orthogonal arrays, which is based on the common industrial use of the different level factors [19].

**Figure 2:**
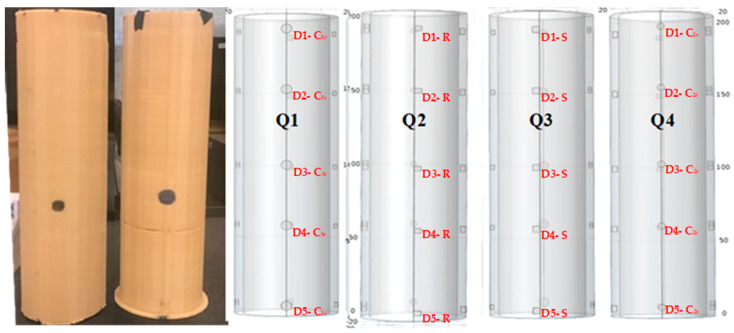
The 3D-printed coupons for the cylinder samples and the COMSOL^®^ model with the four different quadrants.

Two Polylactic Acid Plastic (PLA) samples were designed for validation. Each cylinder has a 2 cm diameter, 20 cm height and 2.5 mm wall thickness. PLA is common in many industries including piping due to many advantages including strength, durability, and low carbon footprints. Each sample is constructed with 20 different embedded defects. As presented in Figure 2, the investigated samples are cylindrical tubes with different densities. The embedded defects are circular, rectangular, and square, while the depth of the defects ranges from 0.3 mm to 1.5 mm (see Table 1).

Vavilov et al. concur that the performance of each NDT technique depends mainly on the context of the inspection routine and its accuracy in terms of detection. For example, the size, shape, type, and depth of defectives sought, affects the quality and performance of the inspection routine. Thus, being able to provide a fast, accurate and reliable inspection while meeting the required quantification level is highly critical [20]. The defects in the cylindrical samples are placed around four quadrants of the sample. Comsol^®^ multiphysics software was used to model and design the specimens. Subsequently, a 3D printer (commercial name Makerbot-Z18^®^, from Makerbot, LLC, New York City, NY, USA) was used to print two cylindrical shaped samples; one with 100% infill density (C100 sample) and the other with 10% infill density (C10 sample). The main objective of printing different samples with different infill densities is to help investigate the defect detection rate, against the scanning routine then conduct a benchmark based on the thermograms.

### 2.1. Line Scan Experiment

**Figure 3:**
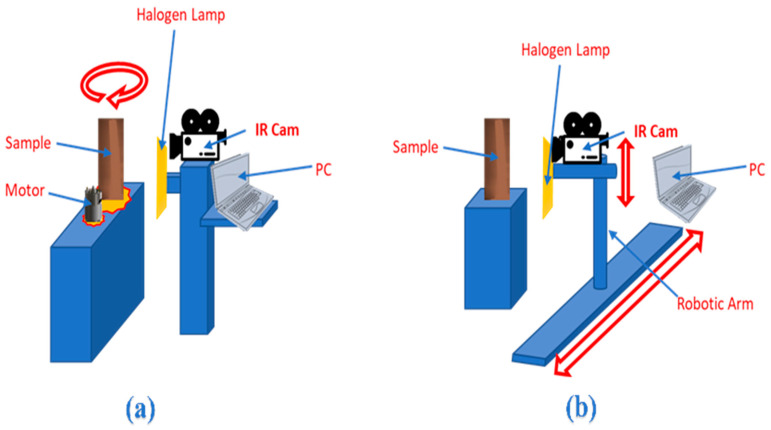
Full view of the experimental setups: (**a**) fixed scan setup and (**b**) line scan setup.

Illustrated in Figure 3, the main experimental setup consists of a thermal camera, heat source, and a PC. A sophisticated infrared camera is put to work to run different scanning thermography experiments. The objective of acquiring thermograms from the infrared camera is to investigate the limitations and strengths with regards to the camera’s sensing sophistication and capabilities. Specifically, the integration time at the array surface or thermal inertia with a cooled micro-bolometer. Furthermore, noise sensitivity is investigated as well as the equivalent temperature difference (NETD) effect on thermal contrast values [21].

The work adopts a FLIR GF309 camera (FLIR Systems, Wilsonville, Oregon, USA) with a 0.025 K (NETD) [22]. FLIR GF309 has an Indium Antimonide cooled Focal Plane Array (FPA), with a Sterling-cycle cooler attached to the imaging sensor to increase the quantum efficiency. Additionally, FLIR GF309 is capable to capture frames with a rate that ranges from 15 to 30 frames per second [23]. For the line and fixed scan experiment, the excitation source used is a 1000-Watt halogen linear heater. The tested samples were thermally excited using the linear heater to detect the defects embedded in the samples. The interactive convective and radiative thermal are defined for the surfaces of the artifacts which dissipate its heat energy into the surroundings. The coefficient of heat convection is assumed to be five W/m^2^. K and 25% of the lamp energy is assumed to be emitted to the PLA artifact. The halogen heater produces continuous energy of up to one Kilojoule with an incident beam with varying angles as the source of the artifacts move [24]. The artificial defects are air pockets; thus, it creates a barrier for the heat diffusion process, which should appear as hot spots in the thermal image.

The testing work aims to collect large datasets from multiple combinations of variables and factors. Explicitly, the study considers three scenarios running on the two different setups which include the line scan setup and the fixed scan setup (Section 3.4). The analyses will be performed on two samples with 40 different defects. Pursuing effective presentation of the methodology, one track is followed to rundown the step-by-step procedure then map it to the rest of the trials. The first step selects the scanning scheme to target the defects at a specific linear-scanning mechanism trajectory. The second step identifies and presents proper image stitching and processing protocol. The third step presents the four main metrics of detectability which include Signal to Noise Ratio (SNR), Analysis of Means (ANOM), Analysis of Variance (ANOVA), and the Aspect Ratio (AR). The foremost metric is the signal-to-noise ratio which compares the level of the desired signal (defect heat) to the level of background (surroundings) noise considering how the response varies relative to the nominal or target value under different noise conditions. The fourth step investigates the detection sensitivity based on two main factors: Factor A, the heat intensity, and Factor B, the scanning speed. The fifth step repeats the analysis based on the below scenarios:

- Scenario 1: fixed depth and varying size
- Scenario 2: fixed size and varying depth
- Scenario 3: dividing the 20 defects into four clusters according to the aspect ratio (AR) calculations.

Toward the end of this section, the data from the remaining trials will be conditioned and used to perform the sensitivity analyses. This analysis will be used to present conclusions and propose recommendations for industrial and future investigations. As stated earlier, there are two different cylindrical samples prototyped for this work. Each cylinder is divided into four quadrants; each quadrant is tested against its lateral surface area, which encompasses four different defects. The first quadrant is placed manually on the inspection table with the line scan inspection traverses at a speed of 23.5 mm/s and a 500 W heating intensity. After the line inspection for the first quadrant is done, the cylinder is rotated clockwise to conduct the same experiment for the second quadrant. The same procedure is applied until the entire area of the cylinder is inspected. The rotation mechanism uses a servomotor controlled with an Arduino system (Boston, MA, USA). Driven with a smart controller, the motor speed and direction are synchronized with the acquisition system to maintain accurate input. Table 2 shows the groupings of the two factors and three levels that are used in all the line scan experiments. Table 3 displays the combinations applied for the line scan.

#### 2.1.1. SNR for Scenario 1 (Fixed Depth and Varying Size)

The infrared camera was put to work to capture the thermal images of a high-density cylinder embedded with defects that have fixed depths and varying sizes. A video is extracted from the thermal sequence and inputted into a stitching code developed in MATLAB^®^ platform (MathWorks Inc., Natick, MA, USA). Afterward, the SNR across the 20 defects in 36 experiments (9 SNRs for 4 quadrant) is calculated using the stitching protocol.

**Figure 4:**
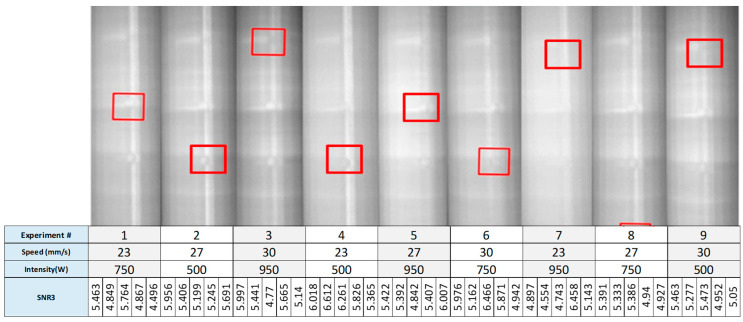
Quadrant 1-Line scan results of the nine experiments, red squares indicate locations of the defects with the highest SNR.

Subsequently, the SNR, ANOM, and ANOVA are computed according to each of the above-mentioned scenarios. There are three different equations that can be applied to find the signal-to-noise ratio, and they are as follows [25]:

(1) $$\mathrm{SNR}=10\;x\log(\frac{{\bar{y}}^{2}}{S_{2}})$$

(2) $$\mathrm{SNR}=-10\;x\log(\frac{1}{n}\Sigma \frac{1}{yi^{2}})$$

(3) $$\mathrm{SNR}=-10\;x\log(\frac{1}{n}\Sigma yi^{2})$$

where n is the number of observations, *y* is the observed data, $\bar{y}$ is the average of observed data and S_2_ is the variation depicted in *y*. The *SNR* for Quadrant 1 of the first cylinder is displayed in Figure 4.

#### 2.1.2. ANOM for C100\Q1

The objective of ANOM calculation is to investigate the effect of each factor and its corresponding level. ANOM assists in distinguishing the least dominant experimental combination and eliminate it from the orthogonal array. Equation (4) was used to calculate the ANOM for the nine experiments, whereas, Equation (5) finds the ANOM for three experiments, representing the investigation of the two main factors: Factor A is heat intensity, and Factor B is the scanning speed.

(4) $$\mu_{1}=\;\frac{\;\sum_{1}^{9}ni}{N\;\;}$$

(5) $$\mu_{2}=\;\frac{\;\sum_{1}^{3}ni}{N\;\;}$$

where *μ*_1_ is the mean of SNRs for the nine experiments, *μ*_2_ is the mean of SNRs for each defect, *N* is the total number of experiments and *ni* is the SNR value for each experiment.

##### ANOM of the Nine Experiments for the First Defect (Defect D1-C_3r_)

Recollecting the SNR values, the ANOM for the first circular defect (D1-C_3r_) can be calculated with nine different combinations of varying speeds and heat intensities, as follows.

$$\mu_{1}=\frac{5.4627+5.9563+5.9971+6.0183+5.4219+5.9755+4.8965+5.3911+5.4626}{9}=5.62\;\mathrm{dB}$$

The calculation is applied for the rest of the defects, with the outcomes tabulated in Table 4. One can observe from the ANOM outcomes that the deviation in the SNR values is less than 16%, which indicates that the procedure is valid for all defects.

##### ANOM with Factor A (Heat Intensity)

The next step validates the assumption that varying heat intensities can affect the level of detection. To test this hypothesis, the ANOM calculations were executed for three trials. The first trial assumes a fixed speed at 23 mm/s and observes the SNR when the heat intensity is increasing. For example, Defect D1-C3_r_ shows the SNR values for (23 mm/s, 500 W), (23 mm/s, 750 W) and (23 mm/s, 950 W) to be 5.4627, 6.0183, and 4.8965, respectively. Observed form the data, increasing the heat to 950 W seems to diminish the heat gradient between defect and the surroundings. The mean for each defect will be compared to the mean of the nine experiments; hence, the mean of the SNR for Defect D1-C_3r_ with fixed 23 mm/s speed is:

$$\mu_{D1-\;C3r}=\frac{{\mathrm{SNR}}_{\left(23,500\right)}+{\mathrm{SNR}}_{\left(23,750\right)}+{\mathrm{SNR}}_{\left(23,950\right)}}{3}=\frac{5.4627+6.0183+4.8965}{3}=5.46\;\mathrm{dB}$$

Table 5 shows the ANOM for the five circular defects. Each defect SNR was tested with three different heat intensities (500, 750, and 950 W). In other words, the heat intensity is tested against varying speeds.

##### ANOM with Factor B (Scanning Speed)

This is the ANOM equation for Factor B where the SNR values correspond to the experiments with 500 W intensity and different speed values. Hence, the SNR values for the same defect (*μ*_D1_-C_3r_) were verified based on the following combinations (500 W, 23 mm/s), (500 W, 27 mm/s) and (500 W, 30 mm/s).

$$\mu_{D1-\;C3r}=\frac{{\mathrm{SNR}}_{\left(23,500\right)}+{\mathrm{SNR}}_{\left(27,500\right)}+{\mathrm{SNR}}_{\left(30,500\right)}}{3}=\frac{5.4627+5.9755+5.3911}{3}=5.61\;\mathrm{dB}$$

The ANOM for the five circular defects is shown in Table 6. Each defect SNR was tested with three different heat intensities (500, 750, and 950 W). In other words, the heat intensity is tested against varying speeds.

#### 2.1.3. Analysis of Variance (ANOVA)

Conducting the ANOVA analysis is beneficial for identifying the dominant controlling factor for each experiment. The ANOVA effect test the proportion of variation by the influential factor over the total variation.

(6) $$ANOVA=\frac{{\mathrm{SS}}_{Factor}}{{\mathrm{SS}}_{T}}$$

(7) $$Total\;Sum\;of\;Squares\;\left(SS_{T}\right)=\sum_{i=1}^{9}{\left(ni-\mu \right)}^{2}$$

where *μ* is the ANOM value, and *ni* is the SNR of each experiment.

The ANOVA of all nine experiments for the first defect (D1-C_3r_) is as shown in the calculation below.

$$\begin{array}{l} SS_{T}={\left(5.4627-5.62\right)}^{2}+{\left(5.9563-5.62\right)}^{2}+{\left(5.9971-5.62\right)}^{2}+{\left(6.0183-5.62\right)}^{2}\\ \;+{\left(5.4219-5.62\right)}^{2}+{\left(5.9755-5.62\right)}^{2}+{\left(4.8965-5.62\right)}^{2}\\ \;+{\left(5.3911-5.62\right)}^{2}+{\left(5.4626-5.62\right)}^{2}=1.204\;\mathrm{dB} \end{array}$$

##### ANOVA with Factor A (Heat Intensity)

The ANOVA is then tested against the three main values that represent Factor A, which calls for sensitivity analysis of the varying heat intensity with fixed speed. The calculation for Defect D1-C_3r_ is shown below. Table 7 lists the ANOVA values for the rest of the defects with regard to Factor A. Additionally, the table shows the percentage of variation observed when considering the variation of heat intensities.

*SS_Factor A_* = 3((5.46 − 5.62)^2^ + (5.59 − 5.62)^2^ + (5.81 − 5.62)^2^) = 0.190 dB

##### ANOVA with Factor B (Scanning Speed)

Similarly, the sensitivity analyses for the ANOVA values are tested with respect to varying speed with fixed heat intensities (Factor B). As shown in Table 8, the percentage of variation caused by Factor B seems to fluctuate, indicating the importance of varying the scanning speed in the detection process. According to the table, the intensity factor is the most dominant compared to the speed factor because it has the highest ANOVA values for all the defects with 17.4%, 32%, 61%, 28%, and 47%. Thus, confirming the results established from the ANOM graphical representation claiming that the intensity factor is the most sensitive.

In the same manner, the rest of the four quadrants are analyzed with SNR, ANOM, and ANOVA analyses. A summary table demonstrates the best combination of factors and levels associated with every defect based on the SNR and ANOM results for Quadrants 2, 3, and 4. The SNR results for Quadrant 2 show that the best heat intensity for all the defect depth is 950 W, whereas the speed varies. Table 9 lists the combinations of heat intensity and speed factors that scored the highest SNR, also known as the best factor combinations.

## 3. Results

**Figure 5:**
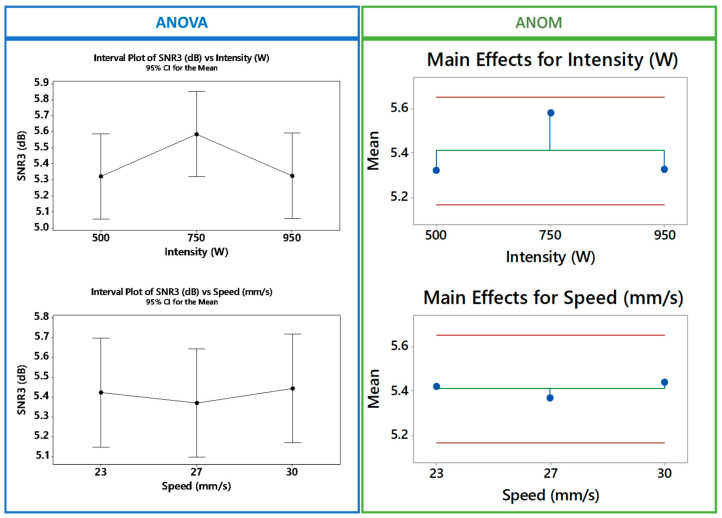
ANOM/ANOVA analyses for Factor A and B using Minitab.

The steps explained in Section 2 are expanded to cover the 40 different defects, which include 20 defects in the low-density sample and 20 in the high-density sample. Testing the impact of the two influential factors (speed and heat intensity) reveals a higher impact of the heat intensity, but the interaction effect is clearly important. The ANOM and ANOVA outcomes shown in Figure 5 show that scanning speed failed to improve defect detectability. On the other hand, the heat intensity factor seems to impose the highest impact on the defect detectability (SNR). The figure shows that 750 W heat intensity increased the SNR to 5.84 dB, while 500 and 900 W heat intensities show poor detectability.

**Figure 6:**
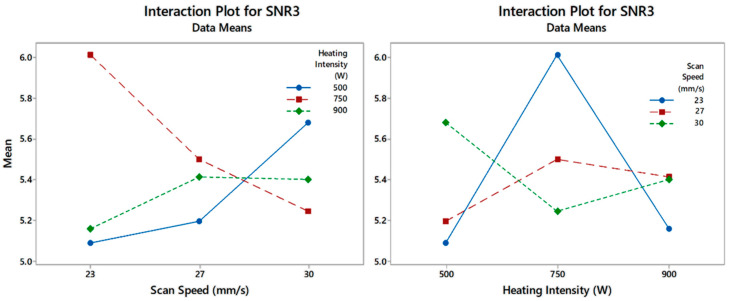
Interaction analyses for scanning speed and the heating intensity.

Further probing of the factors effect shows a clear interaction between the heating intensity and the scanning speed factors (Figure 6). The existence of interaction verifies the implications of this work and upholds the need for simultaneous testing of the factors that affect the detectability of the embedded defects.

### 3.1. Low vs. High Density Sample Results

**Figure 7:**
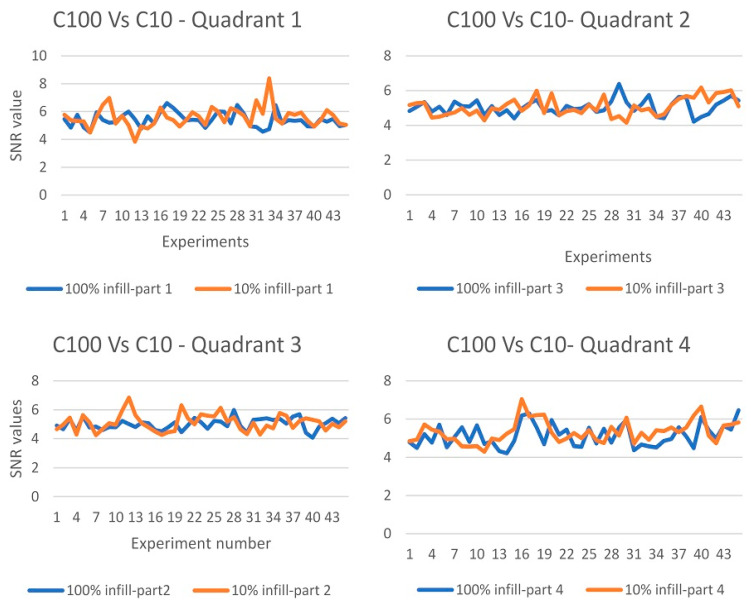
SNR comparison between the C10 and C100 samples.

Endeavoring to test the validity of defect detection with respect to sample density, the analyses were repeated on the light density cylinder (C10). Running the line inspection based on the orthogonal array; 45 readings were observed for every quadrant (9 experiments × 5 defects on each quadrant). The results indicate that some of the defects are not shown as clearly as the full density sample, which is due to the existence of air voids. However, after calculating the SNR value for each defect, it is shown that the maximum difference in the SNR value between the low and the high density samples is very marginal (around 1.8 dB). Figure 7 shows a comparison between the C10 and C100 SNR values for the four quadrants of the two cylinders. This response was not anticipated, but could be justified by the relatively short heating time when running the line scan testing. This is due to the need for rapid testing and swift coverage of more surface area. In the meantime, high infill specimens take more time to heat up, while low infill specimens cool down faster. Consequently, the rate of testing causes the two samples to respond similarly.

### 3.2. Results for Fixed Depth Testing (Scenario 2)

**Figure 8:**
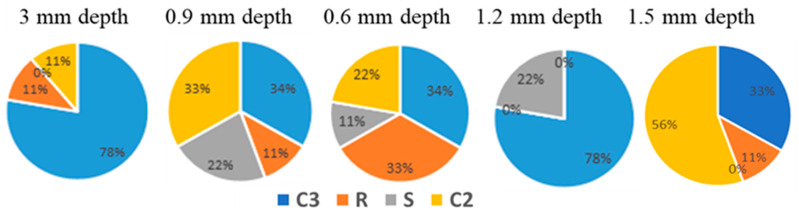
SNR distribution for each experiment with its corresponding defect depth.

The second scenario tests the analysis sensitivities based on SNR values of the nine experiments with fixed defect depth and varying sizes. Thus, a data sheet was created that consists of the SNR values of each circular “C3”, rectangular “R”, squared “S” and circular “C2” per experiment as rows and the columns are the defects’ different depth values. The maximum SNR value for each experiment was found with its corresponding defect size. As shown in Figure 8, defect C3 reached the highest SNR value of 78% of all of the experiments at 0.3 mm depth. In fact, after inputting the percentage values for each defect depth in a pie chart, it appears that the C3 defect had the highest percentage values for all defect depths except for at 1.5 mm. Meanwhile, C2 has the second highest SNR values at the following depths: 0.3 mm, 0.6 mm, and 1.5 mm. As for depths of 0.9 mm and 1.2 mm, the second highest SNR values were for R and S, respectively. The results indicate that despite the different defect depths, overall, a circular shape performs better under the optimized parameters of the simplified form cylinder. It can be concluded that the shape of the defect and defect depth are very important factors that can impact the defect detectability.

### 3.3. Results for Aspect Ratio (AR) Clustering (Scenario 3)

**Figure 9:**
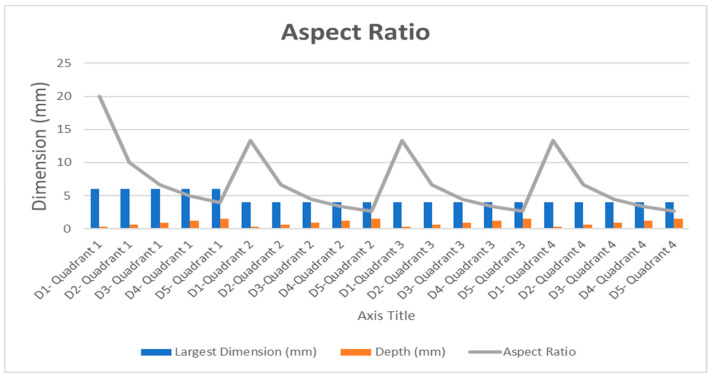
Aspect Ratio Calculations for one cylindrical sample.

The aspect ratio (AR) is an attribute that describes the relationship between the defect’s largest dimension and its depth. Each quadrant of the cylinder contains five defects with the same size, but different depths; thus, the AR value was obtained for all the defects with respect to the size and depth. Figure 9 presents the aspect ratios for one of the cylinders.

Aspect Ratio = (Largest Dimension of the defect (mm))/(Depth of each defect (mm))(8)

The AR data set obtained through the calculations was divided into four main classes: [1–5), [5–10], [10–15], and [15–20]. The 50% of the defects showed to be within the AR ratio range [1–5] and 25% of the defects are in second class, whereas 20% and 5% for classes 3 and 4, respectively. After grouping each defect to a class based on the AR ratio, the lowest SNR value for the defects in each class was selected to conduct the ANOM and ANOVA calculations.

### 3.4. Fixed Scan Experiment Setup

**Figure 10:**
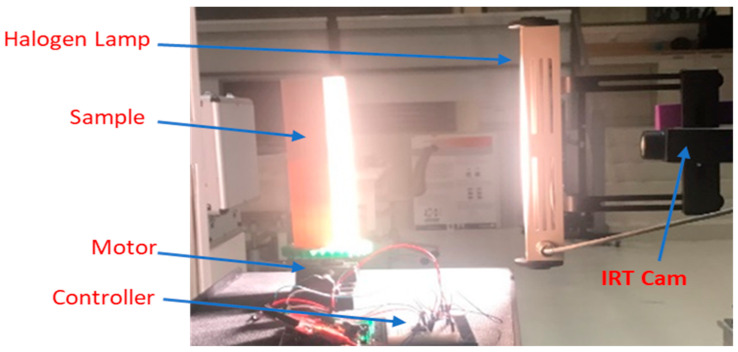
Fixed scan experiment testbed.

As the name implies, the fixed scan experiment considers a fixed camera and heat source with the sample rotating. As presented in Figure 10, the approach uses a high-torque full-rotation servo motor connected to a gear assembly to provide stationary rotation of a base that carries the sample. On the other side, the camera and heat source are mounted on a structure that allows for tilt, pan, and height adjustments. The rotation of the base is controlled by a microcontroller that ensures the correct direction of rotation and accurate rotating speed. The two factors that are manipulated are the rotational speed of the motor and the heating intensity.

**Figure 11:**
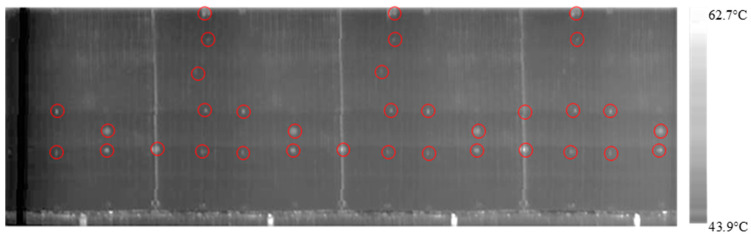
Stitched results of the fixed scan experiment for C100-sample (0.27 rpm & 500 W).

**Figure 12:**
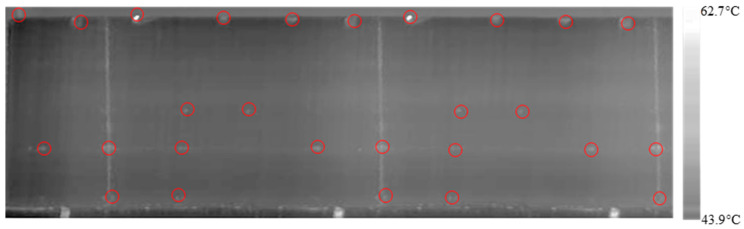
Stitched results of the fixed scan experiment for C10-sample (0.27 rpm & 500 W).

The design of the experiment employed for this experiment employs an L9 standardized table which proposes a total of nine experiments. Initially, the cylinder is placed manually on the inspection table and the servomotor starts rotating the cylindrical sample with 0.27 rpm and 500 W heating intensity. After the first experiment is completed, the sample is cooled down, and then the second experimental combination is initiated. The same procedure is applied until the cylinder is inspected with different factors and level combinations. As shown in Figure 11 and Figure 12, the rotational video of the cylinder obtained from the experiments are flattened using image stitching code in MATLAB^®^. The FLIR GF309 software shows a gradient surface temperature that ranges from 43.9 °C to 62.7 °C.

**Figure 13:**
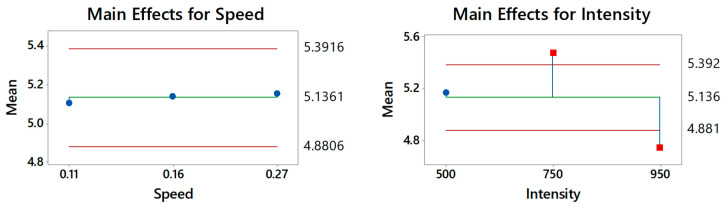
ANOM for the Fixed scan setup.

Figure 13 shows the ANOM for the two influential factors (the heat intensity and scanning speed). The outcomes of the means test 45 defects following the orthogonal array combination of the two factors and three levels that were used in all of the fixed scan experiments based on the L9 standardized table. Comparing the data to those obtained from the line scan experiment, the data seem to reflect negligible disparity. Similarly, the data attest that heat intensity is more influential on detectability than scanning speed.

## 4. Discussion

The results show the ability of the ANOM and the ANOVA to accentuate the variation among the different defects; hence, the defects’ detectability was compared when tested with different approaches. This second section also compares the results of the two scanning setups and their implications on the testing methodology. Furthermore, the results from testing samples with different densities are reviewed aiming to reflect the implications of the established factors on different materials.

### 4.1. ANOM Results

**Figure 14:**
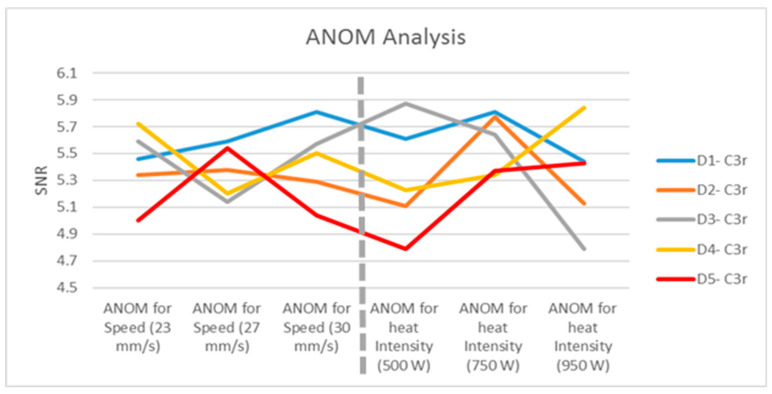
Graphical representation for the ANOM values for the all the defects in one quadrant.

After computing the ANOM value for each of the defects based on the SNR, the ANOM values for all the defects demonstrate that the heat intensity factor is more sensitive than the speed factor, because it has a wide range of values (Figure 14). In addition, the deflection points for defects 1 and 2 are captured in the intensity factor, whereas the rest of the defects’ deflection points lie in the speed factor. The results imply that all of the defects were within the detectable range because of the small difference in the SNR values. However, the heat intensity seems to lose its beneficial effects, as it increases beyond 750 W due to the spreading of the heat causing temperature homogeneity.

### 4.2. Sample Density Comparison Results

After comparing the high-density sample (C100) against the low density one (C10), the results indicate that 50% of the C100 experiments were able to outperform the C10 sample by 5% to 10% more detectability rate. However, this is a low detection difference, which implies that the infill density is not impacted immensely during the fixed scanning routine. In fact, the detectability rates for 40% of the experiments were equivalent for both samples. This verifies that samples with different materials may require different testing settings. The camera was able to show at least 50% of the defects for the C100 sample and 45% for C10. For the lower heating intensity of 500 W, C10 managed to outperform the C100 sample by a range of 5% to 15%. This implies that samples with lower infill densities require less heating intensity when compared to full density samples. Moreover, the maximum percentage difference in the defect detection between the two samples is equal to 20% which implies that the defects in low infill samples can be detected efficiently despite the filling disadvantage. After comparing the sample’s defect detectability rate for both scanning routines, it could be seen that the performance was almost equivalent for both the line and fixed scan routine. Nonetheless, the performance of the C100 was higher than the C10 sample during the line scan with a maximum defect detectability difference of 20%. Consequently, the results indicate a clear relationship between the infill density and the adopted scanning routine.

The clear interaction among factors reinforces the novelty of this work which seeks the effects of power intensity, scanning speed and scanning approach. Meanwhile, four measurable scores are stored in a database that holds the SNR, ANOM, ANOVA and the aspect ratios for each of the factor combinations. The collected database will be used as training data for artificial intelligent IRT control and robotics-controlled pipeline testing.

## 5. Conclusions

This work endeavors to investigate the limitations and sensitivities associated with scanning complex geometry samples using infrared thermography (IRT). A novel approach is used to simultaneously examine three major control factors and observe four measurable outcomes while testing 40 hidden defects with different sizes, shapes, depths, and material densities. 3D-printed cylindrical tubes made of Polylactic Acid Plastic (PLA) were used to validate and compare results. The work employed three 3D-printed samples consisting of a cylindrical tube with 100% infill density (C100), a cylindrical tube with a 10% infill density (C10). A Taguchi DOE was exploited to conduct a thorough investigation of two IRT scanning routines, line scan thermography (LST), and fixed scan thermography (FST). Two main factors were manipulated during the LST experiments. One was the scanning speed of the automated arm and the other is heating intensity. While the FST-controllable parameters considered the rotational speed and heating intensity. The investigations of performance employed Signal-to-Noise Ratio (SNR), while the test sensitivity and best combination of factor levels were determined using Analysis of Means (ANOM) and an Analysis of Variance (ANOVA). The SNR values were very similar, which implies that all the defects were within a detectable range, yet the accuracy of the shape was shown to be different. The ANOM and ANOVA revealed that the most impactful factor for the line scan thermography is the heating intensity, as it relates to the heating source and its coverage to the cylinder. Deeper defects require lower rotational speed to be detected during the fixed scan thermography. It was also observed that the detection of defects of the sample with 100% infill density (C100) performed better than in the one with a 10% infill (C10) sample during the line scan thermography.

Based on these findings, a simulated model is recommended to complement the experimental work. This would allow for more defect scenarios to be captured even within different host materials such as composites. Another recommendation is to use the data found in this work to construct a smart controlling module using open multi-paradigm numerical computing tools to connect the artificial intelligence models to control each parameter of the Inferred Thermography to optimize the nondestructive testing of pipes and related fixtures. Finally, the work recommends enhancing thermograms by applying more sophisticated image processing techniques.

## Figures and Tables

**Table 1:** Descriptions of the 20 different defects employed to test the impact of varying factors on IRT detectability.

**Defect Name**	**Defect Width (mm)**	**Defect Length (mm)**	**Defect Height (mm)**	**Defect Depth (mm)**
D1-R (Rectangular)	3	4	2	0.3
D2-R (Rectangular)	3	4	2	0.6
D3-R (Rectangular)	3	4	2	0.9
D4-R (Rectangular)	3	4	2	1.2
D5-R (Rectangular)	3	4	2	1.5
D1-S (Square)	4	4	3	0.3
D2-S (Square)	4	4	3	0.6
D3-S (Square)	4	4	3	0.9
D4-S (Square)	4	4	3	1.2
D5-S (Square)	4	4	3	1.5
**Defect Name (Circular)**	**Defect Radius (mm)**	**Defect Height (mm)**		**Defect Depth (mm)**
D1-C_2r_	2	2		0.3
D2-C_2r_	2	2		0.6
D3-C_2r_	2	2		0.9
D4-C_2r_	2	2		1.2
D5-C_2r_	2	2		1.5
D1-C_3r_	3	3		0.3
D2-C_3r_	3	3		0.6
D3-C_3r_	3	3		0.9
D4-C_3r_	3	3		1.2
D5-C_3r_	3	3		1.5

**Table 2:** Factors and levels for line scan experiment.

Factors	Level 1	Level 2	Level 3
A: Speed (mm/s)	23	27	30
B: Heat Intensity (W)	500	750	950

**Table 3:** Combinations for line scan.

Experiment #	Speed (mm/s)	Intensity (W)
1	23	500
2	27	750
3	30	900
4	23	750
5	27	900
6	30	500
7	23	900
8	27	500
9	30	750

**Table 4:** ANOM values for nine experiments (C100\Q1).

Defect Name	ANOM of Nine Experiments (dB)
D1-C_3r_	5.62
D2-C_3r_	5.34
D3-C_3r_	5.43
D4-C_3r_	5.47
D5-C_3r_	5.20

**Table 5:** ANOM results when Factor A is tested on the Quadrant 1 of the high-density sample (C100\Q1).

Defect Name	ANOM with Fixed Speed (23 mm/s)	ANOM with Fixed Speed (27 mm/s)	ANOM with Fixed Speed (30 mm/s)
D1-C_3r_	5.46 dB	5.59 dB	5.81 dB
D2-C_3r_	5.34 dB	5.38 dB	5.29 dB
D3-C_3r_	5.59 dB	5.14 dB	5.57 dB
D4-C_3r_	5.72 dB	5.20 dB	5.50 dB
D5-C_3r_	5.00 dB	5.54 dB	5.04 dB

**Table 6:** ANOM results when Factor B is tested on the Quadrant 1 of the high-density sample (C100\Q1).

Defect Name	ANOM with Fixed Heat Intensity (500 W)		ANOM with Fixed Heat Intensity (750 W)	ANOM with Fixed Heat Intensity (950 W)
**D1-C_3r_**	5.61 dB	5.81 dB		5.44 dB
**D2-C_3r_**	5.11 dB	5.77 dB		5.13 dB
**D3-C_3r_**	5.87 dB	5.64 dB		4.79 dB
**D4-C_3r_**	5.23 dB	5.34 dB		5.84 dB
**D5-C_3r_**	4.79 dB	5.37 dB		5.43 dB

**Table 7:** Factor A ANOVA for C100\Q1.

Defect Name	SS_T_ for Factor A (Heat Intensity)	Factor B ANOVA
D1-C_3r_	0.210193749	17.44%
D2-C_3r_	0.828096062	32.73%
D3-C_3r_	1.971164069	61.8%
D4-C_3r_	0.645952376	28.47%
D5-C_3r_	0.752641176	47.45%

**Table 8:** Factor B ANOVA for C100\Q1.

Defect Name	SS_T_ for Factor B (Speed)	Factor A ANOVA
D1-C_3r_	0.190628816	0.190628816/1.204934 = 15.82%
D2-C_3r_	0.010488949	0.010488949/2.530436 = 0.41%
D3-C_3r_	0.383629976	0.383629976/3.184978 = 12.04%
D4-C_3r_	0.408384642	0.408384642/2.268846 = 18%
D5-C_3r_	0.541500082	0.541500082/1.586111 = 34.14%

**Table 9:** Best factor combinations for the defects in the rest of the quadrants.

Quadrant 2	Best Combination	Quadrant 3	Best Combination	Quadrant 4	Best Combination
D1-R	30 mm/s and 950 W	D1-S	23 mm/s and 500 W	D1-C_2r_	23 mm/s and 750 W
D2-R	27 mm/s and 950 W	D2-S	27 mm/s and 750 W	D2-C_2r_	27 mm/s and 750 W
D3-R	23 mm/s and 950 W	D3-S	23 mm/s and 500 W	D3-C_2r_	23 mm/s and 750 W
D4-R	30 mm/s and 950 W	D4-S	30 mm/s and 750 W	D4-C_2r_	27 mm/s and 950 W
D5-R	23 mm/s and 950 W	D5-S	27 mm/s and 750 W	D5-C_2r_	27 mm/s and 750 W
